# Acute and Chronic Effects of Exercise on Appetite, Energy Intake, and Appetite-Related Hormones: The Modulating Effect of Adiposity, Sex, and Habitual Physical Activity

**DOI:** 10.3390/nu10091140

**Published:** 2018-08-22

**Authors:** James Dorling, David R. Broom, Stephen F. Burns, David J. Clayton, Kevin Deighton, Lewis J. James, James A. King, Masashi Miyashita, Alice E. Thackray, Rachel L. Batterham, David J. Stensel

**Affiliations:** 1 National Centre for Sport and Exercise Medicine, School of Sport, Exercise and Health Sciences, Loughborough University, Leicestershire LE11 3TU, UK; J.L.Dorling@lboro.ac.uk (J.D.); L.James@lboro.ac.uk (L.J.J.); J.A.King@lboro.ac.uk (J.A.K.); A.E.Thackray@lboro.ac.uk (A.E.T.); 2 Academy of Sport and Physical Activity, Faculty of Health and Wellbeing, Sheffield Hallam University, Sheffield S10 2BP, UK; D.R.Broom@shu.ac.uk; 3 Physical Education and Sports Science Academic Group, Nanyang Technological University, Singapore 637616, Singapore; Stephen.Burns@nie.edu.sg; 4 School of Science and Technology, Nottingham Trent University, Nottingham NG11 8NF, UK; David.Clayton@ntu.ac.uk; 5 Institute for Sport, Physical Activity and Leisure, Leeds Beckett University, Leeds LS6 3QS, UK; K.Deighton@leedsbeckett.ac.uk; 6 Faculty of Sport Sciences, Waseda University, Saitama 169-8050, Japan; M.Miyashita@waseda.jp; 7 Centre for Obesity Research, University College London, London WC1E 6BT, UK; R.Batterham@ucl.ac.uk; 8 University College London Hospitals Bariatric Centre for Weight Management and Metabolic Surgery, Ground Floor West Wing, 250 Euston Road, London NW1 2PG, UK; 9 National Institute of Health Research University College London Hospitals Biomedical Research Centre, London W1T 7DN, UK

**Keywords:** appetite, energy intake, appetite-related hormones, energy balance, exercise, physical activity, energy compensation, weight control

## Abstract

Exercise facilitates weight control, partly through effects on appetite regulation. Single bouts of exercise induce a short-term energy deficit without stimulating compensatory effects on appetite, whilst limited evidence suggests that exercise training may modify subjective and homeostatic mediators of appetite in directions associated with enhanced meal-induced satiety. However, a large variability in responses exists between individuals. This article reviews the evidence relating to how adiposity, sex, and habitual physical activity modulate exercise-induced appetite, energy intake, and appetite-related hormone responses. The balance of evidence suggests that adiposity and sex do not modify appetite or energy intake responses to acute or chronic exercise interventions, but individuals with higher habitual physical activity levels may better adjust energy intake in response to energy balance perturbations. The effect of these individual characteristics and behaviours on appetite-related hormone responses to exercise remains equivocal. These findings support the continued promotion of exercise as a strategy for inducing short-term energy deficits irrespective of adiposity and sex, as well as the ability of exercise to positively influence energy balance over the longer term. Future well-controlled studies are required to further ascertain the potential mediators of appetite responses to exercise.

## 1. Introduction

The prevalence of overweight and obesity has reached epidemic levels in multiple countries [1]. Elevations in body adiposity substantially diminish health-related quality of life via an increased risk of cardio-metabolic, neuromuscular, cancerous, and psychological co-morbidities [2]. Furthermore, increased health-care costs and lost productivity due to rising obesity levels leads to considerable economic strain [3]. One therapeutic intervention for the prevention and management of obesity is exercise. There is compelling evidence that exercise can assist in the prevention and management of numerous cardio-metabolic, neuromuscular, osteopathic, and psychological conditions, yet its intrinsic effect on obesity is more contentious [4,5,6]. Individuals who exercise regularly are less likely to be overweight or obese, and exercise interventions are effective in provoking weight loss and facilitating weight loss maintenance, particularly when combined with dietary interventions [7]. Nevertheless, some individuals experience large reductions in body fat whilst others exhibit little change [8,9]. It therefore remains a contemporary challenge for researchers to identify the individual factors that underlie the variability in body fat responses to exercise regimens.

Further to elevations in energy expenditure and fat oxidation, cross-sectional evidence suggests that exercise may influence energy balance through variations in energy intake [10,11]. Accordingly, a body of evidence has investigated the appetite and energy intake responses to exercise interventions in an attempt to delineate the relative importance of appetitive changes in exercise-induced weight management. This includes studies that have examined appetite and energy intake variables in response to single bouts of exercise and chronic exercise training interventions. More recently, many studies have concurrently examined changes in appetite-related hormones to better understand the mechanisms that are postulated to affect appetite and energy intake alterations after exercise. A collective feature of these studies is the substantial variability in appetite, appetite-related hormone and energy intake responses to exercise [12,13]. This may occur because of the multiplicity of factors that influence appetite and energy intake [14], but differences in individual characteristics and behaviours, including adiposity, sex, and habitual physical activity, may also modulate appetite responses to exercise. Given the importance of appetite and energy intake in energy homeostasis, an improved understanding of the key individual characteristics and behaviours that adjust appetitive measures after exercise has important implications for weight management. 

Therefore, the aim of this review is to provide an overview examining the modulating effect of adiposity, sex, and habitual physical activity on the appetite, energy intake and appetite-related hormone responses to acute and chronic exercise interventions. Furthermore, this review will highlight the limitations associated with this body of work and consider avenues for future research. The review is narrative in nature, with the intention of identifying and discussing the most relevant evidence representative of trends in this field of research, rather than providing a systematic account of studies in the area. Although this is not a systematic review, studies that examined the acute and chronic effects of exercise on appetite-related measures were identified from the author’s libraries and computer-based databases including PubMed and Google Scholar. Studies with varying methodologies and robustness were considered, but all were critically appraised and discussed to clarify the status of this field of research.

## 2. Appetite, Energy Intake and Appetite-Related Hormone Responses to Exercise Interventions

Evidence examining the acute and chronic effects of exercise on appetite, energy intake, and appetite-related hormone responses has been reviewed extensively [15,16,17,18,19]. A brief summary of this body of work is presented in the next section to provide a context for understanding the modulating effects of adiposity, sex, and habitual physical activity on appetite responses to exercise. 

### 2.1. Acute Exercise

A plethora of studies have examined the appetite-related responses during and after single bouts of continuous aerobic exercise, with the majority of these studies performed in lean, physically active males. Overall, these studies indicate that subjective feelings of appetite are transiently suppressed during exercise performed at or greater than 60% peak oxygen uptake (VO_2_ peak); a phenomenon termed exercise-induced anorexia [20,21,22,23,24]. This acute change has been reported in response to resistance exercise but is less marked [25,26] and is not observed consistently [27,28]. Appetite perceptions typically return to resting control values within 30 to 60 min of exercise cessation [23,25,29,30,31,32], and they do not stimulate changes in energy or macronutrient intakes on the day of aerobic exercise [20,29,33,34,35,36,37] and resistance exercise [25,26,28,36,38]. 

On a meal-by-meal basis, an array of episodic hormones secreted from a diffuse population of endocrine cells within the digestive tract modulate feelings of satiation and postprandial satiety [39]. These include the orexigenic hormone acylated ghrelin, and the anorexigenic signals peptide tyrosine tyrosine (PYY), glucagon-like peptide-1 (GLP-1), and pancreatic polypeptide (PP). Single sessions of exercise have consistently been shown to suppress circulating concentrations of acylated ghrelin during aerobic exercise at intensities above 60% VO_2_ peak [23,24,25]. The effect of resistance exercise on acylated ghrelin concentrations appears less definitive, with limited evidence reporting either a suppression [25] or no change [27,36] in concentrations, in response to the exercise stimulus. Simultaneously, elevations in satiety hormone concentrations including PYY, GLP-1, and PP have been reported during aerobic exercise bouts [27,29,30,36,40,41,42], though again, these changes appear to be less profound during resistance forms of exercise [25,27,36]. These hormonal fluctuations are short-lived, typically returning to resting control values in the hours after exercise [22,23,29,30,41].

### 2.2. Chronic Exercise

Although fewer exercise training studies with appetite-related outcomes have been conducted, these investigations are crucial to determine the effectiveness of exercise as a lifestyle strategy for weight control. However, evidence investigating the chronic effects of aerobic exercise on appetite parameters is largely conflicting. Some studies have shown that subjective appetite in the fasted state is increased after aerobic exercise training [43,44,45], whereas others have reported no change [46,47,48], or even a reduction in appetite [49]. Furthermore, aerobic exercise training has been shown in some studies to have negligible effects on energy or macronutrient consumption [9,46,50,51], while others have demonstrated that energy intake is reduced [52] and that protein intake is elevated [48] after training. Limited evidence has examined appetite responses to resistance training, with no change in fasting or postprandial appetite reported after a 12 week regimen [49]. 

In spite of inconsistent findings, it has been suggested that chronic exercise alters the sensitivity of the appetite control system by balancing the increased drive to eat with an improved satiety response to a meal [43,45]. This is supported by evidence suggesting that appetite and ad libitum energy intake are reduced after consuming a high- but not low-energy density meal in individuals undergoing a structured exercise training program [12,46,48]. 

As key regulators of the nutritional state, many studies have examined changes in two tonic appetite suppressants, leptin and insulin, in response to exercise training. These generally report reductions in leptin after aerobic and resistance exercise training [47,49,53], whereas the findings for insulin are more variable, with some studies demonstrating a reduction [47,50], and other studies demonstrating no change [48,49,54] in concentrations after exercise training. Further ambiguity revolves around the long-term changes in episodic peptides from the digestive tract. A small collection of studies have shown an increase in concentrations of acylated ghrelin, PYY, GLP-1, and PP [45,47,50], but other evidence suggests that negligible differences exist after resistance exercise training [49]. 

## 3. Body Adiposity

Despite the systemic rise in obesity levels in recent decades, there is substantial variability in whole-body adiposity between individuals within westernised societies [55]. A myriad of factors modulate energy intake and energy expenditure with varying magnitudes, and they could contribute to the wide variability in adiposity levels observed between individuals. In this regard, body fat, in itself, may modulate appetite, appetite-related hormones, and food intake after exercise. This is particularly evident with episodic appetite-related hormones, most notably acylated ghrelin, PYY, and GLP-1, as fasting concentrations are lower and postprandial changes are blunted as body fat increases [56,57,58]. Likewise, individuals with overweight or obesity display elevated concentrations of leptin and insulin [57,59]. As such, exercise studies with appetite, energy intake, and appetite-related hormone measures have been performed in participants with a wide range of adiposity levels.

### 3.1. Acute Exercise

In line with evidence in individuals who are lean, exercise-induced anorexia has been reported in studies recruiting individuals with overweight or obesity [60,61]. However, findings are inconsistent. Studies in participants with overweight or obesity (body mass index (BMI) ≥ 25.0 kg·m^−2^) reported no change in subjective appetite perceptions during acute aerobic exercise at moderate and high intensities [27,62,63], or acute resistance exercise [27]. In addition, individuals with overweight and obesity do not demonstrate increased appetite after exercise [27,63,64,65]. Negligible changes in energy and macronutrient intakes have also been reported between control and exercise trials on the day of exercise [63,64,65], although a reduction in energy intake at a single-course porridge meal has been observed in individuals with overweight after high-intensity exercise [62]. Precise reasons for discrepancies in appetite and energy intake findings are challenging to pinpoint, but variations in meal composition and/or timing as well as the exercise protocol adopted are likely to contribute.

Akin to studies in individuals who are lean, reductions in acylated ghrelin and elevations in PYY, GLP-1, and PP have been reported concurrently with a suppression of appetite during aerobic exercise in individuals with overweight/obese [27,60,61]. One study additionally found that total PYY, total GLP-1, and PP did not change after resistance exercise in men with overweight (average BMI: 29.9 kg∙m^−2^) [27], which is in line with work performed in lean men (average BMI: 23.7 kg∙m^−2^) [36]. However, despite showing a reduction in acylated ghrelin in response to aerobic exercise, two studies found that moderate- and high-intensity cycling did not alter circulating PYY concentrations in individuals with overweight/obese [62,63]. This contrasts with evidence in lean individuals who display a greater exercise-induced suppression of appetite, and an increase in PYY concentrations at higher exercise intensities [66]. Subsequent appetite-related hormone assessments after exercise have also yielded conflicting findings. Specifically, Unick et al. [64] demonstrated that GLP-1 concentrations were lower 1 h after a single bout of moderate-intensity walking in women with overweight/obese (BMI 25.0–34.9 kg∙m^−2^), yet Holliday and Blannin [61] showed recently that GLP-1 was elevated 1 h after low-volume sprint interval cycling in individuals with overweight or obesity (BMI 25.0–34.9 kg∙m^−2^). Other studies have also demonstrated that PYY and PP concentrations are unchanged in the hours after exercise in individuals with overweight or obesity [62,63].

It is very likely that the heterogeneity in methodologies and outcome measures among these studies contributes to some of the variance in findings. This can, however, be attenuated when groups with differing body adiposity are compared directly within the same protocol. In the earliest instance of this type of study, Kissilef et al. [67] compared the consumption of a yoghurt test meal provided 15 min after a 40 min cycling bout performed at either 30 or 90 watts in nine women who were lean (average BMI: 22.1 kg∙m^−2^) and nine women with overweightness (average BMI: 27.8 kg∙m^−2^). The authors reported that grams of yoghurt consumed was lower after the bout of cycling performed at 90 watts compared with cycling at 30 watts in the women who were lean but was not significantly different between trials in the women with overweight [67]. Although the reduced energy intake may be a continuation of exercise-induced anorexia, these findings may suggest that individuals who are lean exhibit different post-exercise food consumption patterns compared with individuals with overweight. In contrast, Ueda et al. [30] reported that individuals with overweight/obese (BMI 26.0–34.6 kg∙m^−2^) displayed a greater reduction in energy intake at a single-course pasta meal 1 h after moderate-intensity exercise than a group of individuals who were lean (BMI 19.1–24.7 kg∙m^−2^) [30]. Furthermore, the authors observed a similar elevation in total PYY and GLP_7–36_ concentrations up to 1 h after exercise in both groups, which did not coincide with any exercise-induced alterations in appetite or acylated ghrelin [30].

**Figure 1:**
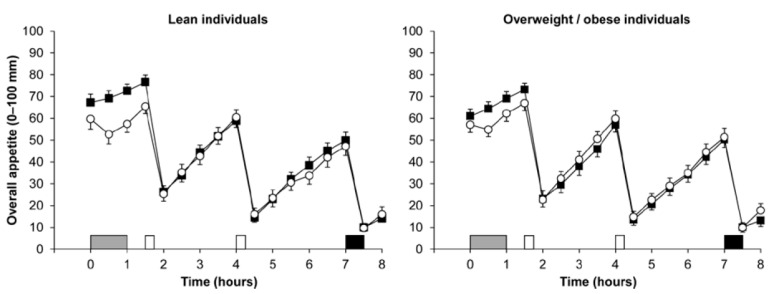
Overall appetite perceptions in individuals who were lean (*n* = 22; left panel) and those with overweight/obesity (*n* = 25; right panel) during the control (■) and exercise (○) trials. Exercise involved 60 min treadmill exercise at a 59% peak oxygen uptake. Data are mean ± SEM. A grey rectangle indicates exercise, open rectangles indicate standardized meals and black rectangle indicates an ad libitum buffet meal. Data from Douglas et al. [33]. © Springer Nature. Reproduced through Creative Commons licence.

To further investigate the effect of exercise on appetite responses in groups varying in adiposity status, Douglas et al. [33] recently measured appetite, appetite-related hormones and ad libitum energy intake both during and for seven hours after 60 min of moderate-intensity treadmill exercise in individuals who were lean (BMI 19.6–24.5 kg∙m^−2^) and individuals with overweight/obese (BMI 25.3–35.4 kg∙m^−2^) [33]. The authors reported that the exercise bout transiently suppressed perceptions of appetite to a similar extent in both adiposity groups (Figure 1), and did not alter energy or macronutrient intake in response to an ad libitum buffet meal provided 6 h after exercise cessation in either group [33]. Although this contrasts with previous findings, the disparity in ad libitum energy intake may be related to differences in meal timings or variations in meal composition. With regard to appetite hormones, Douglas et al. [33] reported that exercise-induced changes in total PYY and GLP-1 may be modulated by adiposity status, with the group with overweightness/obesity exhibiting a greater elevation in total GLP-1 after exercise, whereas the group that were lean demonstrated a greater exercise-induced increase in total PYY. Although these changes may represent differences in appetite-related hormone regulation between individuals varying in adiposity status, these differences were small [56]. Further studies are needed, particularly in isoforms that are more implicated in appetite regulation such as active PYY (PYY_3–36_) and active GLP-1 (GLP-1_7–37_). On the contrary to total PYY and total GLP-1, Douglas and colleagues reported that concentrations of acylated ghrelin were not modified after exercise in the participants who were lean, or in those with overweightness/obesity [33]. This may reflect the lower exercise intensity in this study (59% VO_2_ peak) compared with previous investigations reporting a suppression in acylated ghrelin at exercise intensities above 60% VO_2_ peak [22,23,24,25]. Nevertheless, another study reported a similar magnitude of reduction in acylated ghrelin in individuals who were lean, or in individuals with obesity after cycling exercise to exhaustion, albeit the group with obesity exhibited lower concentrations of acylated ghrelin at baseline [68].

Collectively, despite some inconsistencies in the literature, the balance of evidence suggests that acute exercise-induced changes in appetite and energy intake are similar between individuals who are lean and individuals with overweight/obese. Future research is required to examine the influence of adiposity on appetite measures after resistance exercise, since these changes have primarily been investigated in individuals who are lean. Further studies are, moreover, needed to clarify potential disparities in appetite-related hormones using groups with distinctively different BMIs and body fat percentages. Careful grouping of cohorts through body fatness would also allow researchers to examine the mechanisms underlying appetite-related hormone differences between individuals who are lean and those with overweight/obesity. This could include the changes in insulin sensitivity and glycemic control, given that impairments in these parameters have been implicated in explaining the lower fasting concentrations and blunted postprandial responses of appetite-related hormones in individuals with overweight/obesity [69,70].

### 3.2. Chronic Exercise

The majority of exercise training studies with appetite-related measures have been performed in individuals with overweightness and obesity. This body of work suggests that exercise training increases fasting hunger and postprandial satiety [43,71], and may improve the coupling between energy intake and energy expenditure in response to food intake [12,43,45]. This was shown by King et al. [43], who demonstrated that 12 weeks of supervised aerobic training in individuals with overweight and obesity (average BMI: 31.8 kg∙m^−2^) augmented fasting hunger but also increased the satiety response to a fixed meal. As an exception, Martins and colleagues investigated appetite and energy intake responses to six weeks of aerobic training performed four times a week in individuals who were active and lean (average BMI: 22.7 kg∙m^−2^) [48]. Appetite perceptions measured before and after the consumption of low and high energy preloads (246 vs. 607 kcal, respectively) were not altered in response to exercise training [48], yet 24 h energy intake, measured through a single buffet lunch and self-reported food diary, was lower post-training after the provision of the high energy preload [48]. Conveniently, these results can be compared with a study that applied a similar paradigm before and after a 12 week exercise training intervention in individuals with overweight and obesity (BMI 27–35 kg∙m^−2^) [46]. This study similarly demonstrated that energy intake in the 24 h after consumption of the high energy preload was lower post-training but was unchanged in response to exercise when the low energy preload was consumed. Taken together, the investigations by Martins and colleagues suggest that physiological sensitivity to high energy preloads may be improved after exercise training, independent of body adiposity [46,48].

With regards to appetite-related hormones, four weeks of moderate-intensity cycling at 55% VO_2_ peak has been shown to reduce concentrations of leptin and increase concentrations of GLP-1 in sedentary men (average BMI 25.6 kg∙m^−2^) [47]. In another study, Pil-Byung et al. [53] reported that concentrations of leptin and total ghrelin were reduced after an 8 week programme of combined resistance and aerobic training in inactive subjects (BMI 25.5 kg∙m^−2^). Although this contrasts previous findings demonstrating no change [49] or an increase [45] in acylated ghrelin concentrations after a chronic exercise intervention, it may be related to the divergent properties of the two ghrelin isoforms, with total ghrelin comprising levels of both acylated ghrelin and desacylated ghrelin. Further studies should attempt to distinguish between acylated and desacylated ghrelin during hormonal analysis as desacylated ghrelin may oppose the orexigenic and metabolic functions of the acylated isoform [72].

Notwithstanding the changes in appetite-related hormone concentrations, Pil-Byung et al. [53] and Morishima et al. [47] found that exercise lowered body weight and abdominal fat-mass, respectively, which could mediate the post-training changes in appetite-related hormones. With this considered, it is possible that individuals with higher pre-training adiposity display greater changes in appetite-related hormones that are proportional to reductions in body fat. This is well illustrated by Gibbons and colleagues who demonstrated recently that individuals with overweightness and obesity who lost more weight after a 12 week aerobic exercise intervention displayed an elevated postprandial rise in GLP-1 and total PYY, and a greater suppression in acylated ghrelin compared to those who lost less weight [73]. Adding support to the notion that changes in body fat are key to changes in appetite hormones, one study showed that greater weight loss in response to high-intensity interval training was associated with greater post-training increases in fasting acylated ghrelin [71]. Martins et al. [71] did not, nevertheless, find these relationships with concentrations of PYY_3–36_ or GLP-1, and other studies have shown unchanged fasting and postprandial concentrations of acylated ghrelin [49], PYY_3–36_ [50], and GLP-1 [45], despite reductions in body weight after aerobic exercise training programs. Guelfi and colleagues also showed that a reduction in fat mass after resistance exercise training did not coincide with changes in acylated ghrelin, PYY, or PP concentrations in men with overweightess/obesity [49]. Additionally, another study in individuals with obesity reported training-induced elevations in fasting and postprandial PP concentrations in the absence of body fat loss [54]. Reasons for these discrepancies are unclear, and further studies are needed to clarify the variations in appetite-related hormones after exercise training that could be modulated by the body fat of participants. In these studies, efforts should be made to define adiposity status with precise body fat percentage measures alongside BMI. This is key, as BMI is intrinsically unable to distinguish fat mass from fat-free mass, which could lead to individuals being wrongly defined as lean, overweight or obese [74]. Moreover, given visceral adiposity may suppress ghrelin concentrations [75], future studies should also consider the modulating effect of body fat distribution by defining groups, based on waist circumference or abdominal adiposity.

## 4. Sex

Scientific investigations that analyse the exercise-induced changes in appetite, appetite-related hormones and energy intake are less abundant in women than men. Inclinations to preferentially study men in exercise and appetite research are likely to be related to cyclical changes in sex hormones in women that engender fluctuations in appetite, appetite-related peptides, and energy intake during different phases of the menstrual cycle [76]. Indeed, evidence suggests that energy intake, plasma GLP-1, and insulin are suppressed during the follicular phase of the menstrual cycle, whereas total PYY concentrations are lower during the luteal phase [76,77]. Studies on the appetitive changes after exercise have, however, been performed in women, especially in light of research, suggesting that women may compensate for the exercise-induced energy deficit over the longer term in order to preserve higher body fat stores compared to men [78]. The interested reader is directed to a recent review for a more comprehensive insight on this topic [79], but the aim of this section is to consolidate these findings and supplement them with recent evidence.

### 4.1. Acute Exercise

The consensus of evidence demonstrates that women exhibit equivalent appetite responses to men after a single bout of exercise. Specifically, an early study demonstrated that energy intake at a buffet meal was not significantly different in 11 females when measured 15 min after a period of rest, short duration exercise, long duration exercise, or intermittent exercise [80]. Most ensuing studies conducted in single groups of women have also shown no compensatory increase in appetite or energy intake to account for the energy expended through exercise in the short term [64,65,81,82]. Exercise-induced anorexia and transient changes in appetite-related hormones have also been demonstrated in response to exercise in women, with reductions in acylated ghrelin, and elevations in PYY and GLP-1 concentrations reported during and in the hours after exercise [34,83]. However, these findings are not universal with some studies, suggesting that women do not demonstrate a suppression in appetite [64,84] or changes in appetite-related hormones including acylated ghrelin, PYY and GLP-1 [64,85,86] in response to acute exercise stimuli. Furthermore, another study conducted exclusively in women reported an increase in acylated ghrelin and a tendency for higher energy intake after 60 min of exercise at 70% VO_2_ peak [86]. It is possible that consumption of a standardised breakfast prior to exercise in the latter study [86] elevated PYY and lowered acylated ghrelin concentrations, and it may have diminished the transient exercise-induced responses that are often seen with these peptides.

**Figure 2:**
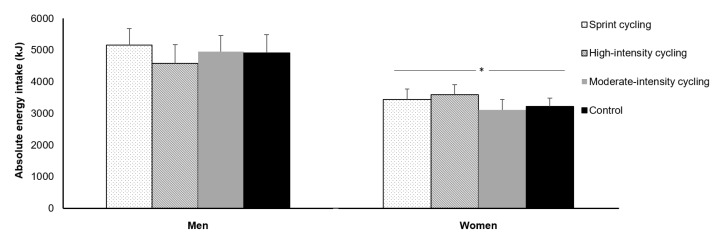
Absolute energy intake at an ad libitum buffet meal in the control, moderate-intensity, high-intensity intermittent and sprint interval cycling trials in 11 men and nine women. Data are mean ± SEM. * Significant difference between men and women. Data from Panissa et al. [87]. © RightsLink. Reproduced with permission.

Direct comparisons of acute exercise-induced changes in appetite parameters between the sexes have been explored in six studies to date [34,38,83,87,88,89]. An important control feature included in these studies was that female participants completed all trials in the follicular phase of the menstrual cycle to minimise any potential confounding effects on the measured appetite parameters [34,83,87,88,89]. A single bout of vigorous-intensity exercise (70% VO_2_ peak) has been shown to elicit a similar transient suppression of appetite perceptions in both men and women but did not provoke any changes in ad libitum energy or macronutrient intakes on the day of exercise in either sex, despite males displaying greater absolute intakes [34,88]. Recent studies have also examined whether sex modulates appetite responses to exercise performed at different intensities and using varying modalities. Hazell et al. reported a comparable suppression in appetite between men and women after a single bout of moderate-intensity cycling (30 min, 65% VO_2_ peak) or sprint interval cycling (6 × 30 s ‘all-out’ sprints, 4 min recovery) [83]. This supports the findings of another study demonstrating equivalent appetite responses between the sexes after acute bouts of work-matched continuous moderate-intensity cycling (60% maximal attained power), high-intensity intermittent cycling (1 min at 100% maximal attained power, 1 min recovery) or all-out cycling sprints (60 × 8 s ‘all-out sprints, 12 s recovery) [87]. The authors also reported no change in ad libitum energy intake in response to the various exercise protocols in either sex, albeit absolute energy intake was higher in men than women (Figure 2) [87]. A recent study also demonstrated that self-reported 48 h energy/macronutrient intakes were not different between work-matched high-intensity intermittent and low-intensity continuous cycling bouts in either sex [89]. However, in contrast to studies adopting aerobic and intermittent exercise protocols, one study showed that ad libitum energy intake was higher 75 min after resistance exercise compared to aerobic exercise in men but was similar between the exercise protocols in women [38]. This implies that sex may modulate food intake changes after resistance exercise, but these findings were derived from a small sample and require further verification.

In addition to appetite and energy intake measures, many of these aforementioned studies examined potential sex-based differences in appetite-related hormone responses to exercise [34,83,87,88,89]. Despite evidence of higher fasted concentrations of acylated ghrelin at baseline in women compared with men [33,34], the acute exercise-induced suppression in acylated ghrelin appears to be similar between the sexes [34,87,88]. However, the potential modulating effect of sex on satiety hormone responses to acute exercise appears to be inconclusive. Whilst Panissa and colleagues demonstrated no effect of exercise on PYY_3–36_ [87], Hagobian et al. documented that PYY_3–36_ concentrations were increased after exercise in women, but not in men [88], whereas Hazell et al. found that total PYY levels immediately after sprint interval cycling were only elevated in men [83]. Although total PYY and PYY_3–36_ concentrations are correlated, differences in biochemical processing and analysis required for these isoforms could be implicated in these variations [90]. Furthermore, Hazell et al. reported that moderate-intensity and sprint interval cycling augmented total GLP-1 concentrations in women, but not men [83], whereas another study demonstrated no effect of high-intensity intermittent or continuous low-intensity cycling on total GLP-1 concentrations in men and women [89]. Despite these inconsistencies in the current literature, there is little evidence to suggest that a single exercise bout differentially affects appetite, energy intake, or the appetite-related hormone milieu in men and women. Similar appetite responses between sexes after exercise are observed, even when the greater energy expenditure and energy intake of men are considered [34,87,88,89,91].

### 4.2. Chronic Exercise

The appetite and appetite-related hormone variations in men and women after exercise training are important, particularly as women are postulated to exhibit greater compensatory elevations in energy intake that may result in lower weight loss after exercise training regimens compared to men [78]. Although many exercise training studies have combined the data for men and women together precluding any direct comparisons, a few studies have directly examined potential sex-based differences. One research group measured food intake through weighed food records, and indicated that women, but not men, partially compensated for the exercise-induced energy deficit (~30% of energy expended through exercise) after seven days of daily supervised exercise [92,93]. Conversely, in a study that measured food consumption during a 16 day supervised exercise intervention period in six men and six women, Whybrow et al. showed that average daily energy intake was partially increased in men (~30% of energy expended during exercise) but not women [94]. There was, however, a tendency for higher energy intake in women after exercise intervention, and a greater sample size may have resulted in a significant finding. Interestingly, these sex-related variations occurred without significant losses of body weight in men or women, suggesting any changes in food intake may occur independent of body weight changes after short-term exercise regimens.

In one study where equivalent reductions in percentage body fat occurred between the sexes, fasting hunger and postprandial satiety after a fixed meal were increased to a similar extent in 107 men and women with overweight or obesity after 12 weeks of aerobic exercise training [44]. The authors also reported that total 24 h energy and macronutrient intakes from an individualised fixed-energy breakfast, fixed-energy lunch, ad libitum evening meal and an ad libitum snack box were not influenced by exercise training in men and women [44]. Of note, the exercise intervention in this study was designed to induce an energy expenditure of 500 kcal per session in both sexes, and all exercise bouts were supervised to ensure that the target exercise stimulus (70% maximum heart rate, five sessions per week) and energy expenditure were controlled [44]. These features of the study design are important, as previous research suggests a higher exercise-induced energy deficit may stimulate greater compensatory elevations in energy intake [95]. However, after a comparable 12 week intervention, Caudwell and colleagues [12] reported that the intake of high energy density foods was higher and lower in men and women, respectively. This was accompanied by a greater exercise-induced reduction in body fat in the men, which may have contributed to the divergent energy intake changes reported [12]. Similarities in the design of these two studies make it challenging to explain variations in findings, but it does emphasise the difficulties in accounting for the factors impacting energy balance over a training intervention. For instance, although the stringent laboratory-based measures of energy intake and exercise energy expenditure in these studies should be commended [12,44], it is possible that free-living changes in these parameters outside the laboratory may have influenced the study outcomes in men and women. Measuring the impact of these factors is fastidious and standard energy intake measures may be insufficient to detect these small variations. Future studies may benefit from utilising the laws of thermodynamics to calculate energy intake from changes in body energy stores and energy expenditure measures, though this requires accurate and precise measures of energy expenditure (e.g., doubly labelled water) and body composition [96]. 

Evidence examining between-sex differences in appetite-related hormones in response to a period of exercise training is sparse. Nevertheless, fasting leptin and insulin concentrations were significantly reduced in women but not men, after 12 weeks of exercise training, despite no training-induced changes in body fat in either sex [97]. Similarly, a more recent study reported that women with overweight and obesity displayed a greater reduction in insulin and a greater elevation in acylated ghrelin than men in the postprandial state, after a short four-day exercise program [91]. Although Hagobian et al. [91] reported no differences in appetite or fasting leptin in men and women, these two studies support the suggestion that women may exhibit divergent changes in the appetite-related hormone milieu compared with men, in response to an energy deficit that is induced by exercise. However, more research is necessary to examine the potential sex-based differences in the response of tonic and episodic appetite-related hormones to more prolonged exercise training interventions before definitive conclusions can be drawn. Current evidence is also restricted to studies adopting aerobic exercise training interventions, and therefore, research investigating the potential sex-based differences in appetite responses to other exercise modalities, such as resistance training, represents another avenue for future scientific enquiry.

## 5. Habitual Physical Activity and Exercise

Physical activity and exercise aids weight control and improve metabolic health [98]. In spite of the influence of purposeful exercise on appetite, appetite-related hormones, and food intake, varying amounts of habitual physical activity may modify appetitive responses [18]. Evidence from cross-sectional research has indicated that inactive individuals have impaired energy intake regulation, which may facilitate a positive energy balance and consequential weight gain [10,99]. This section will discuss recent research that has explored differences in appetite-related responses to exercise interventions between habitually active verses inactive individuals.

### 5.1. Acute Exercise

Although most acute exercise studies have recruited recreationally active individuals, some research has been conducted in inactive and extremely active individuals. Evidence in this regard suggests both active [25,28,36] and inactive [38] groups of participants do not demonstrate compensatory changes in energy intake after resistance exercise. Resistance exercise does not likewise induce any changes in satiety appetite-related hormones in active [25,36] and inactive [27] individuals. With aerobic exercise, one study reported that appetite and energy intake were not affected by a single bout of walking in individuals who habitually exercise less than two times a week for 30 min [64]. At the opposite end of the physical activity spectrum, Howe et al. [81] demonstrated that single bouts of moderate- and high-intensity exercise transiently suppressed subjective appetite and acylated ghrelin, and increased PYY_3–36_ and GLP-1 in a group of endurance-trained female athletes habitually exercising on at least five days a week. However, the evidence is far from consistent and other studies report conflicting findings in individuals with substantially different activity levels. A recent study showed that PYY_3–36_ was unaffected and ad libitum energy intake was reduced in response to a 20 km run in a group of endurance athlete runners [100]. Additionally, Larsen-Meyer and colleagues reported that acylated ghrelin was increased after 60 min running at 70% VO_2_ max in trained runners, but was unchanged after 60 min walking at a similar relative intensity in a group of habitual walkers [86]. The authors also reported that ad libitum energy and macronutrient intakes after exercise were not altered in the runners, whereas ad libitum energy, protein, and fat intakes were higher after the walking bout in the group of walkers [86]. This is in agreement with Finlayson et al. [101], who showed that individuals with augmented energy intake and a higher preference for energy-dense foods after 50 min of vigorous-intensity cycling had lower self-reported levels of habitual physical activity. Compensatory energy intake after exercise in less active individuals could speculatively be associated with metabolic and substrate oxidative variability during exercise. Hopkins et al. [102] demonstrated that carbohydrate oxidation during exercise was positively correlated with energy intake at a test meal provided 60 min after a bout of cycling performed at 70% of the maximum heart rate. Given that aerobic training and fitness reduces relative carbohydrate oxidation and increases dependence on fat as a fuel source during exercise [103], these findings may offer one possibility for explaining why inactive individuals may be more likely to counter the energy expended during single bouts of exercise. It may alternatively be that individuals with greater levels of physical activity are more informed on dietary guidelines and make healthier food choices [104,105]. Equally, in response to the activity that they are more accustomed to, physically active individuals may consciously not increase energy intake shortly after exercise, as their energy deficits are offset by elevated energy intake over a prolonged period. Physically inactive individuals, in contrast, may perceive exercise as a greater novel challenge that is significantly less enjoyable [106,107]. Therefore, they may be more likely to acutely augment energy intake as a reward in lieu of habitually altering food intake over the long term.

Nonetheless, Jokisch et al. demonstrated that hunger was suppressed in response to an exercise bout completed at 65–75% maximum heart rate in a group of inactive men, but not in a group of active men [37]. The authors also found that energy intake at a cold-item buffet meal was only suppressed in response to exercise in the inactive males [37]. Conversely, other studies indicate that energy intake variations are negligible between active and inactive groups. Charlot and Chapelot reported that 24 h food and macronutrient intakes were similar between high and low activity groups after a 60 min cycling bout at 70% VO_2_ peak [108]. Underlining the contradictory results, one research group has reported mixed trends in experiments examining the influence of a moderate-intensity cycling bout on self-reported food intake in active and inactive groups three days after exercise [109,110,111]. Indeed, in males, Rocha et al. [111] demonstrated that active individuals compensated more for the exercise-induced energy deficit on the day of exercise, yet the inactive group exhibited greater energy intake on the third day after exercise [111]. Likewise, a follow-up study demonstrated that self-reported energy intake the day after exercise was only reduced in an inactive group of women [109], whereas energy intake changes after exercise were not different between active and inactive women in another study [110]. The authors opined that with females consuming oral contraceptives solely in the former study [109], hormonal medication may influence post-exercise responses in active and inactive females [110]. However, it may be that the high random error associated with self-reported measures of food intake contribute to these mixed findings [112].

### 5.2. Chronic Exercise

Given exercise training studies typically recruit individuals who are inactive at baseline, the modulating effect of habitual physical activity after an exercise training intervention is difficult to delineate. However, cross-sectional studies are available that have measured fasting and postprandial appetite and energy intake in individuals with varying levels of habitual physical activity. Gregersen et al. [113], for example, measured subjective appetite ratings in response to a standardised evening meal in 178 men and women (aged 20 to 60 years) stratified into those who trained four or more times a week and those who participated in less than four exercise sessions a week. The authors reported that those with greater habitual physical activity exhibited reduced satiety and increased hunger compared to the less physically active group [113]. Although this study did not standardise energy intake for more than four hours before the evening meal, these findings are supported by another study demonstrating that active individuals had higher self-reported energy and protein intake compared with inactive individuals [114]. Furthermore, the active individuals displayed higher levels of energy intake when the ad libitum test meal was not preceded by a yoghurt preload [114]. These studies jointly suggest that physically active individuals may consume higher quantities of energy to offset the energy expenditure induced by habitual physical activity.

Other studies have compared energy intake responses between individuals of varying physical activity levels after manipulating the energy content of preloads provided before an ad libitum meal. The earliest study adopting this paradigm demonstrated that active individuals reduced their energy intake after a high compared with low energy preload whereas energy intake was similar after both preloads in inactive individuals [115]. This supports the findings of a recent study that assessed energy intake after high and low energy preloads in a cohort of individuals stratified into tertiles based on objectively-derived accelerometery data [116]. In their analysis, Beaulieu et al. [116] reported that the reduction in energy intake after a high-energy preload was augmented in the two tertiles with the highest levels of habitual moderate-to-vigorous physical activity relative to the tertile with the lowest levels. These results suggest that individuals with higher levels of moderate-to-vigorous physical activity may demonstrate improved regulation of energy balance [116]. Nonetheless, the interaction between physical activity levels and energy compensation after different energy preloads has only been investigated in response to a single laboratory-based meal. Therefore, further studies that directly compare physically active and inactive groups that adopt differing energy preloads and prolonged energy intake assessments are needed to elucidate the modulating effect of habitual physical activity on prolonged energy intake patterns. 

Very few studies have investigated the effect of habitual physical activity on appetite-related hormone responses. In one example, Lund et al. [117] studied the appetite-related hormone changes to a fixed liquid meal in a group of endurance-trained athletes who exercised more than three days a week over several years, and a group of inactive individuals who had not exercised in the last six months. The authors reported that concentrations of fasting acylated ghrelin and GLP-1 were elevated in the active group compared with the inactive group, while the active group also showed higher concentrations of GLP-1 in the postprandial state [117]. In contrast, total PYY and PP were not statistically different between training groups, though it is possible these null findings were due to insufficient statistical power [117]. Another study also demonstrated that plasma levels of insulin were lower in individuals who exercised for a minimum of 150 min a week for at least two years, compared with inactive individuals [114]. These differences in appetite-related hormones could be related to the increased skeletal muscle GLUT4 levels in active individuals that increase the capacity for muscle glucose uptake and lower postprandial blood glucose levels [118,119]. Equally, however, differences could be related to distinct variations in body fat, as the active groups had markedly lower levels of adiposity that are likely to, at least partially, mediate variations in the appetite-related hormones. 

A challenge of these studies is in distinguishing the individual impact of self-reported physical activity and objectively measured aerobic fitness on these variables as both are inexorably linked. This is pertinent to other studies discussed in this review as all studies that measured VO_2_ peak alongside self-reported habitual physical activity demonstrate that individuals with greater levels of habitual physical activity have higher aerobic fitness levels [37,86,101,108,109,111,114,117]. Further studies are also needed to verify findings using objective measures of physical activity. Systematic and random errors are frequent in self-report measures, due to poor activity recall and frequent overestimation of physical activity levels [112]. While recent work has utilised objective technology [116], the majority of studies classify individuals based on the method of physical activity recall. Therefore, it remains difficult to fully ascertain the magnitude of habitual physical activity that confers changes to appetite-related parameters. Likewise, it is not known if different physical activity modalities, such as aerobic or resistance, modulate these measures. This represents a key avenue for further study since higher levels of fat-free mass associated with traditional resistance training have been posited to increase appetite and food intake through rises in resting metabolic rate [12,120]. 

## 6. Implications and Areas of Future Study

On balance, the current evidence suggests that neither adiposity nor sex modulate appetite, appetite-related hormone, and energy intake responses to acute and chronic exercise, but individuals who are physically active appear to have an improved appetite sensitivity, which may facilitate long-term energy balance. This suggests that exercise should be promoted as a universal method of inducing an acute energy deficit, and engaging in regular physical activity may promote a closer coupling of energy intake and energy expenditure over the longer term. The consensus of evidence reviewed here supports the ongoing promotion of exercise as a weight management tool for both facilitating the maintenance of a healthy weight, and for reducing body weight and fatness. Nonetheless, from a practical standpoint, different groups may need to modify exercise strategies to attain sufficient energy deficits that optimise the effectiveness of exercise in weight management. For example, individuals with lower fitness may need to exercise for longer durations and/or at greater relative intensities to achieve similar energy deficits to lean individuals with high physical fitness. Similarly, females may require longer or more intense exercise bouts to induce a given energy deficit than males, though this deficit may not need to be as great, due to the habitually lower energy intake in females [12,33,34,87,88,89,91].

It is unquestionable that further studies are required to elucidate how adiposity, sex, and habitual physical activity affect appetite, energy intake, and appetite-related hormone responses to acute and chronic aerobic and resistance exercise. In these studies, efforts should be made to directly compare divergent adiposity, sex, and habitual physical activity groups within the same study design whilst controlling for potentially confounding variables that may modify outcome measures. In addition, a variety of inter-individual physiological factors should be examined in relation to exercise and appetitive parameters. First, given the literature associating certain common genetic polymorphisms with body adiposity through differences in appetitive measures [121], the appetite, energy intake, and appetite-related hormone responses to exercise should be investigated in variations of obesity and appetite-related genes. Chief amongst these are single nucleotide variations of the *fat mass and obesity associated gene* [122]. The rationale for investigating the appetite-related responses to exercise in variants at this gene is strong as carriers of the ‘obesity-risk’ variants display greater postprandial appetite (impaired satiety), energy intake and acylated ghrelin concentrations [123,124]. Consequently, future experiments are needed to examine if the appetite and body mass differences between individuals carrying different variants at this gene can be offset by exercise. Second, more studies are needed in groups with varying fat-free mass. It is particularly important to investigate the interactive impact of exercise modality and body composition alterations on appetite-related measures, since resistance training stimulates greater elevations in fat-free mass compared to traditional aerobic training [49]. In combination with research comparing groups with different levels of adiposity, studying the effect of varying levels of fat-free mass on post-exercise appetite and appetite-related hormones would improve understanding of the relative impact of different body tissues on appetite regulation and energy balance. Third, it is necessary to examine appetite- and appetite-related hormones after acute and chronic exercise across a spectrum of ages. Weight gain typically occurs throughout adulthood with body weight loss from the age of 65 years and onwards [125]. Though metabolic and musculoskeletal mechanisms are implicated, changes are partly linked to alterations in appetite [126]. This is relevant later in life as elderly individuals display a decline in energy intake, which may be related to a concomitant elevation in leptin and cholecystokinin, and a reduction in ghrelin [127].

## 7. Methodological Issues

Although several patterns in appetite, energy intake, and appetite-related hormone responses after exercise have been identified in this review, there are numerous methodological limitations within the field that will undoubtedly contribute to the variability in findings. Specifically, many studies do not have suitable comparison groups that are adequately powered and carefully chosen in order to pinpoint the influence of inter-individual variables. This is important as many of the features discussed are inexorably linked, which means distinct groups frequently differ by more than one inter-individual variable. Accordingly, studies should aim to recruit well-matched groups who have similar biological characteristics and behaviours other than the independent variable of interest. Although the precise matching of groups is challenging, such procedures will better ascertain the modulating effect of adiposity, sex and habitual physical activity *per se* on appetite-related dependent variables in response to exercise. 

An extra source of inconsistency in the studies discussed is the wide-ranging methods used to measure energy intake. Some investigations administer self-report food diaries in free-living conditions, but these have been criticised due to consistent over- and under-estimations of energy intake that can amount to hundreds of calories per day and can affect statistical power within small-scale studies [112,128]. While other studies carefully measure weighed food intake within a controlled laboratory environment, the external validity of these measures can be questioned with the erratic food intake frequently reported [13]. Studies that seek to assess energy and macronutrient intake after exercise in different groups of individuals may thus benefit from limiting the selection of food items and familiarising participants to their respective buffet meal. Additionally, despite being in their infancy, future research may benefit from novel devices that assess food intake through swallowing monitors, camera pictures, and wrist motion detectors [112]. 

Scrutiny should also be placed on the collection, biochemical processing, and analysis of blood samples for the determination of appetite-related hormone concentrations. Venepuncture and cannulation procedures can cause stress that leads to rising levels of PYY_3–36_ and a decrease in subjective appetite [90]. Evidence indicates that this response can be mitigated through adequate familiarisation to blood sampling methods and/or acclimatising participants to the cannula for a 45–60 min period before sampling for appetite-related hormones [90]. This could have ramifications for several studies cited in this review that assess appetite-related hormones via venous blood sampling, and that do not sufficiently habituate their participants to a cannula or invite participants for multiple trials within a study. Moreover, modified appetite-related hormone concentrations due to apprehension with blood sampling could contribute to the inconsistency with findings in this field. While there is likely to be substantial variability in anxiety responses between participants, investigators should consider habituating participants to venous blood sampling methods and they should examine the effect of trial order on appetite-related hormones during repeated-measure designs [90]. In addition to sampling, efficient processing of blood samples is critical, as many appetite-related hormones are volatile and concentrations can be rapidly altered ex vivo by various endogenous esterases and proteases [90,129]. As a result, studies determining the concentrations of these hormones typically need to rigorously treat blood samples with enzyme inhibitors and to, with certain peptides such as acylated ghrelin, acidify plasma prior to analysis. On occasion, however, exercise and appetite studies measuring these hormones do not appear to adhere to recommended treatment procedures. It is possible that this sub-optimal treatment of samples could contribute to the contrasting findings that are reported, regarding exercise-induced changes in appetite-related hormone concentrations [63,87,88]. Further to the immediate management of collected blood, it is likely that discrepancies in appetite-related hormones exist because of varying specificity and sensitivity of the assay used [17]. Therefore, future studies measuring appetite-related hormone responses to exercise should seek, where possible, some consistency with assay kits and procedures [17].

## 8. Conclusions

The present review suggests that changes in perceptions of appetite, energy intake, and macronutrient composition in response to acute and chronic exercise stimuli are not modulated by levels of body adiposity or sex. Although further research is required to extend the evidence base, individuals with higher levels of habitual physical activity may exhibit improved sensitivity of the appetite control system through better compensatory adjustments for the energy content and density of food. There are, nonetheless, multiple discrepancies in appetite-related hormone findings, which make it difficult to summarise how adiposity, sex, and habitual physical activity modulate exercise-induced changes in these hormones. An improved understanding of the individual factors that modulate appetite, appetite-related hormone, and food intake responses to exercise may help to explain the individual variability in body weight changes after exercise, and to facilitate the development of more efficacious weight management interventions.
